# LIFE Med Hiss: An innovative cohort design for public health

**DOI:** 10.1016/j.mex.2018.12.007

**Published:** 2018-12-18

**Authors:** Martina Gandini, Cecilia Scarinzi, Stefano Bande, Giovanna Berti, Luisella Ciancarella, Giuseppe Costa, Moreno Demaria, Stefania Ghigo, Chiara Marinacci, Antonio Piersanti, Gabriella Sebastiani, Ennio Cadum

**Affiliations:** a University of Torino, Department of Clinical and Biological Science, AOU San Luigi Gonzaga, Regione Gonzole 10, 10043, Orbassano, Turin, Italy; b Environmental Epidemiological Unit, Regional Environmental Protection Agency, Piedmont Region, Via Pio VII 9, 10135, Turin, Italy; c Air Quality Unit, Regional Environmental Protection Agency, Piedmont, Via Pio VII 9, 10135, Turin, Italy; d Laboratory of Atmospheric Pollution, ENEA-Bologna Research Center, Via Martiri di Monte Sole 4, 40129, Bologna, Italy; e Regional Epidemiology Unit, ASL TO3 Piedmont Region, Via Sabaudia 164, 10095, Grugliasco, Italy; f Department of Epidemiology, Lazio Regional Health Service, Rome, Italy; g National Institue of Statistics, Rome, Italy

**Keywords:** LIFE Med Hiss project approach, Air pollution, Epidemiological surveillance, Long term studies, National Health Interview Surveys, Exposure assessment

## Abstract

The aim of MED HISS methodology was to test the effectiveness of a low-cost approach to study long-term effects of air pollution, applicable in all European countries. This approach is potentially exportable to other environmental issues where a cohort representative of the country population is needed.

The cohort is derived from the National Health Interview Survey, compulsory in European countries, which has information on individual lifestyle factors. In Life Med Hiss approach, subjects recruited have been linked at individual level with health data and have been then followed-up for mortality and hospital admissions outcomes. Exposure values of air pollution (PM2.5 and NO_2_) have been assigned using national dispersion models, enhanced by the information derived from monitoring station with data fusion techniques, and then upscaled at municipality level (highest level of detail achievable for the Italian Survey). Results for mortality have been used to test the effectiveness of this methodology and are encouraging if compared with European ones.

The advantages of this technique are summarized below:

• It uses a cohort already available and compulsory in European countries
• It uses air quality modelling data, available for most of the countries
• It permits to implement versatile environmental surveillance systems

## Graphical abstract

**Figure:**
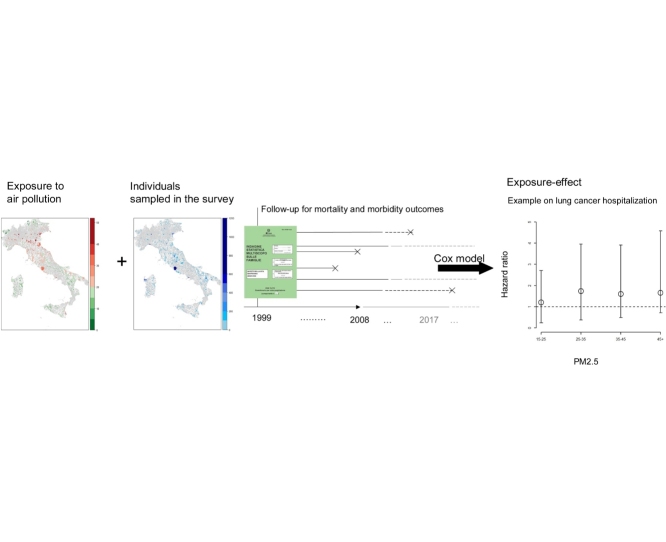

**Specifications Table**

**Table:** 

Subject Area	*Environmental Science*
More specific subject area:	*System to measure adverse health effect of air pollution using a cohort derived from National Health Interview Survey*
Method name:	*LIFE Med Hiss project approach*
Name and reference of original method	*NA*
Resource availability	*NHIS* (https://www.cdc.gov/nchs/nhis/index.htm) *MINNI* (http://www.minni.org/?set_language=en) *BRACE* (http://www.brace.sinanet.apat.it/) *European Air Quality Dataset Airbase* (http://www.eea.europa.eu/) *Corine Land Cover* (https://land.copernicus.eu/pan-european/corine-land-cover) *R* (https://cran.r-project.org/bin/windows/base/) *SAS* (https://www.sas.com/it_it/home.html)

## Method details

LIFE MED HISS project aimed at consolidating the knowledge base for the development, assessment, monitoring and evaluation of environmental policy and legislation, by setting up a low-cost European surveillance system of long-term effects of air pollution based on cohorts recruited using National Health Interview Survey (NHIS) data already available.

A large number of epidemiological studies have linked long-term concentrations of air pollution to mortality, but relatively few studies focused on the long term effects of particulate (PM10 and PM2.5) or NO_2_ on hospital admissions. As mortality risk factor, in 2015, ambient PM2.5 was classified as the fifth-ranking. Most of the cohort studies were carried out in USA, where both the characteristics and susceptibility of the population exposed and the composition of air pollution mixture can be different from European context. Several cohorts are available in Europe, as the national English cohort (812,063 patients aged 40–89 years as reported by [1]), the Danish Diet, Cancer, and Health (DCH) cohort (57,053 people from Copenhagen or Aarhus aged 50–65 years, as reported by [2]) or the London cohort, which studied also combined effects of noise and air pollution [3].

The cohort we recruited is based on the National (Italian) Health Interview Survey, which is compulsory as in all European countries and has information on individual lifestyle factors. This cohort has been followed-up for mortality and morbidity. For exposure assessment it was possible to assign to each individual the exposure value to air pollution (PM2.5 and NO2), derived from national dispersion models, improved with monitoring station using data fusion techniques, and then upscaled at municipality level.

### Health data

**Fig. 1:**
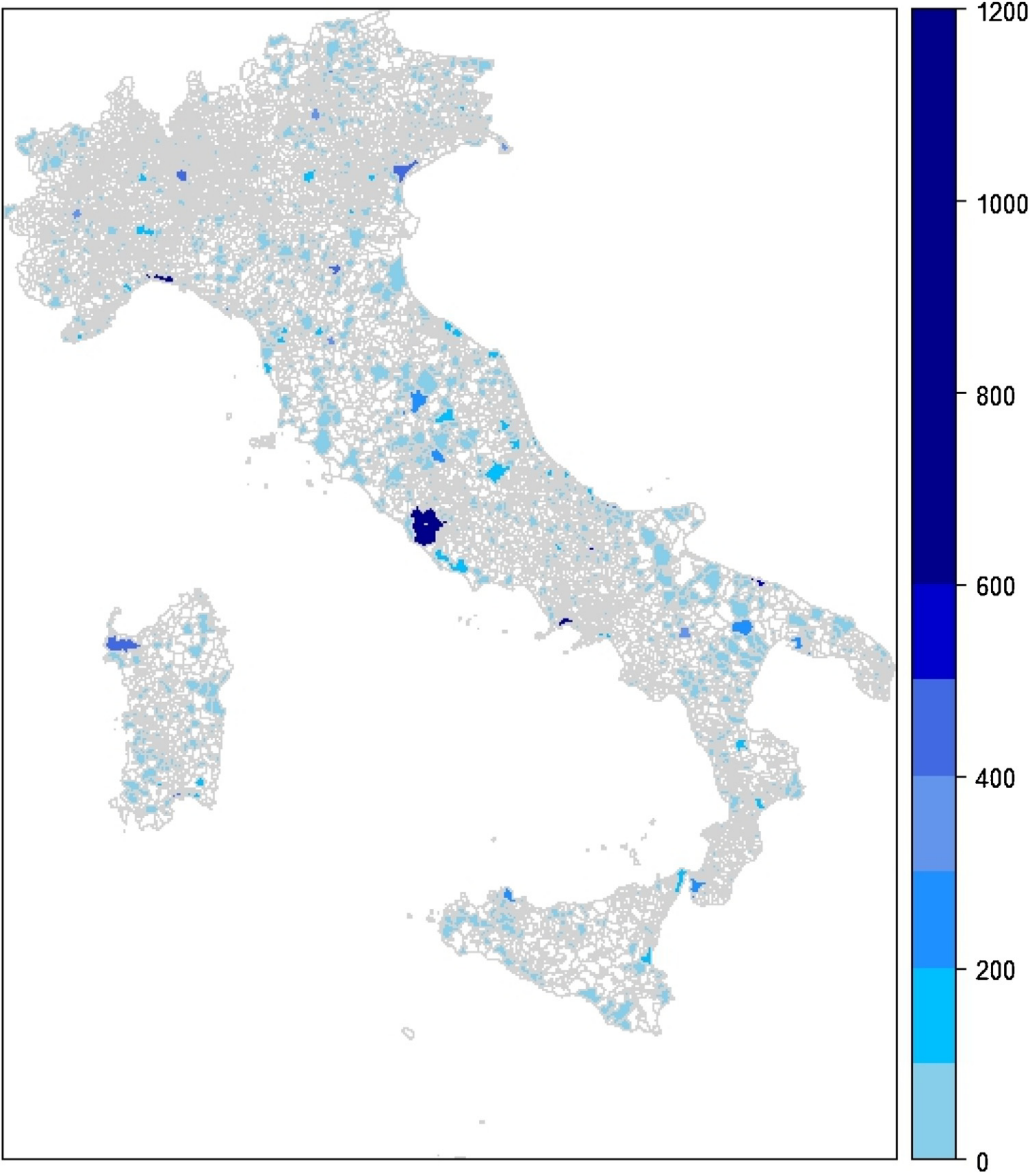
Number of sampled subjects for each municipality.

The follow-up is based on the sample included in the 1999–2000 NHIS, carried out by the National Institute of Statistics (ISTAT), linked to mortality and hospital records [4]. The original NHIS sample consists of 140,011 individuals belonging to 52,232 sampled families resident in 1449 municipalities in the whole national territory (Fig. 1). The NHIS contains, beside others, individual information on age, gender, occupational and educational status, marital status, residence, body mass index (BMI), smoking habit and physical activity. Information on alcohol was not available in the survey, while information on diet was limited to particular conditions (e.g. macrobiotic diet) and therefore could not be used to assess lifestyles. In Fig. 1 there is a map with the number of subjects sampled in each municipality.

To perform record linkage, the personal data from the paper format of family status of the interviewed people made available by ISTAT were recorded. At the end of the registration and control operations, an archive was obtained with the personal data of 139,991 individuals. This archive was linked to the cohort of the interviewees (140,011). Using the information from both archives, the Fiscal Code necessary for the record linkage with the other sources was calculated. Of the 140,011 respondents, 128,818 individuals were linkable with full or partial fiscal code, representing 92% of the HIS sample. A record linkage has been carried on for mortality and hospital admissions with the ISTAT national archive of the causes of death. The tax code was used as record linkage key. When missing in the death certificate, tax code was imputed based on the reported personal information. The codification of causes of death and hospitalization has been performed using an automatic procedure: the rejects that cannot be automatically processed (about 20% of the total) are manually coded. The underlying cause is selected according to international rules from the World Health Organization, reported in the International Classification of Diseases, ninth revision (ICD-9), for all cases up until 2002 and in International Classification of Diseases, tenth revision (ICD-10), for all cases from 2003 onwards. Only for interviewed individuals with complete fiscal code (126,601) a record linkage between 1999–2000 NHIS and hospital discharges archives of the Ministry of Health has been performed. Hospital admissions with fiscal code were available only starting from year 2001.

Record linkage has been the most critical point in the setting up of the project. Two participating countries (Spain and France) could not apply this methodology due to privacy legislation and had to carry on another methodology [5]. Slovenia instead, took advantage of this project to improve privacy regulation. By the end of the project they have been able to enrol a Slovenian cohort and this kind of methodology will be applied when an adequate number of follow-up years will be reached.

To evaluate the completeness of the record linkage between the cohort and the data kept in the death records’ national archive, the number of deaths observed in the sample was compared with the expected number. The number of expected deaths has been obtained by multiplying the Italian age and sex-specific mortality rates observed in each follow-up year with the actual number of individuals of the cohort at risk of death for each year, for the specific sex and age (person-years). A similar procedure has been applied to evaluate record linkage with hospital admissions. Both checking operations succeded in positive results. Moreover, descriptive statistics on the original sample and that one resulting from the follow-up have been computed to avoid possible distortion in the representativeness of the Italian population.

We investigated six causes of mortality: non-accidental (ICD-9 code 001–799, ICD-10 code A00-R99), death for Cardiovascular Diseases (CVD) (ICD-9 code 390–459, ICD-10 code I00-I99), diseases of the respiratory system (ICD-9 code 460–519, ICD-10 code J00-J99), neoplasm excluding lung cancer (ICD-9 code 140–239 except 162, ICD-10 code C00-D48 except C33–C34), lung cancer (ICD-9 code 162, ICD-10 code C33-C34) and diseases of the nervous system (ICD-9 code 320–359, ICD-10 code G00–G99). We decided to investigate neoplasm excluding lung cancer to consider separately a cause of death which is strongly associated with air pollution according to literature.

We excluded from the analyses non-accidental causes and repeated hospitalizations for the same subject (with the exceptions of myocardial infarction, angina pectoris and low respiratory tract infection (LRTI)). For each cause, we considered only the main cause of hospitalization out of six available, and, for each subject, only the first hospital admission, excluding all subsequent hospitalization for the same cause and for the same subject. This criterion permits to have a good approximation to an incidence measure. An exception to this rule was done for diabetes and chronic obstructive pulmonary disease (COPD), which were searched among all six codes. A second type of exception was done for myocardial infarction, angina pectoris and LRTI: for the first two causes, a second event that occurred after 28 days or more from the first episode was considered as a new event [16], whereas for LRTI the time elapsed from the first episode to consider it as a new event had to be 90 days or more.

Since the topic was relatively new considering hospitalizations as outcome, we selected a wider number of causes. Firstly, the following main group of causes have been analyzed: disease of the circulatory system (ICD-9 code 390–459), heart diseases (ICD-9 code 390–429), cerebrovascular diseases (ICD-9 code 430–438), diseases of the respiratory system (ICD-9 code 460–519), all neoplasm but lung cancer (ICD-9 code 140–239, with the exception of 162), mental, behavioral and neurodevelopmental disorders (ICD-9 code 290–319), diseases of the nervous system (ICD-9 code 320–359). Secondly, we investigated the following specific causes: lung cancer (ICD-9 code 162), bladder cancer (ICD-9 code 188), kidney cancer (ICD-9 code 189), diabetes (ICD-9 code 250), Parkinson’s disease (ICD-9 code 332), Alzheimer’s disease (ICD-9 code 331), myocardial infarction (ICD-9 code 410), angina pectoris (ICD-9 code 413) atherosclerosis (ICD-9 code 440), LRTI (ICD-9 code 466, 480–487), COPD (ICD-9 code 490–492, 494, 496), asthma (ICD-9 code 493), and miscarriage (ICD-9 code 634).

**Fig. 2:**
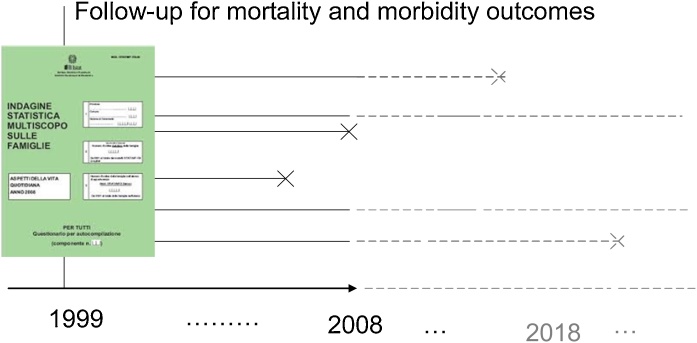
Graphical representation of the cohort together with the follow-up.

In Fig. 2 there is a graphical representation of the follow-up scheme. An individual which took part to the NHIS may experience one of the outcomes of interest during the follow-up (until year 2008), after the follow-up, or may not have experienced the event to the present day.

Due to the nature of the LIFE MED HISS project, variables included in the model as covariates are harmonized, according to the standard developed within the European EUROTHINE project [6], ensuring a high degree of comparability with other European surveys, also considering levels chosen for categorical variables. Covariates included in the model are: gender, educational level, marital status, occupational status, smoking habit, physical activity, BMI, type of municipality.

### Exposure to air pollution

Due to the nature of the NHIS, which has individuals sampled both in rural and urban areas of the whole national territory, the best choice to assign exposure data may be the use of modelling data. Since in health data it was possible to know only residence’s municipality, with no information on address or zip code, a comparable national coverage has been needed for air quality data.

The Italian exposure model considers data coming from the modeling system MINNI (National Integrated Modelling system for International Negotiation on atmospheric pollution), developed and validated by ENEA and the Italian Ministry of the Environment Land and Sea [7,8]. MINNI includes an atmospheric chemical transport model (CTM) fed by emission data (national emission inventory of anthropogenic sources, biogenic VOCs, sea-salt, natural dust) and meteorological prognostic fields. MINNI outputs for PM2.5 and NO_2_ were provided for years 1999, 2003, 2005 and 2007 at a spatial resolution representative of urban background pollution levels (4 × 4 km^2^) at hourly resolution ([7]b). The CTM model included in the MINNI modelling system is called FARM [13,15] and the version used in the study is the 3.1.12.

To reduce model uncertainty, a data fusion technique is used to combine MINNI concentration fields and pollutant observations. Thus, it is possible to provide a more realistic representation of pollutant spatial distribution. Due to the nature of the health data, only background stations have been considered to integrate CTM models. Observed data introduced in the dispersion model outputs are retrieved from the Italian regional environmental agencies BRACE database (The national system: http://www.brace.sinanet.apat.it/) and from the European Air Quality dataset AirBase (The European air quality database: http://www.eea.europa.eu/ data-and-maps/data/airbase-the-european-air-quality-database-7). Only monitoring stations with less than 30% missing data have been considered in the assimilation procedure.

Therefore, a Kriging with External Drift (KED [9], and [10]) procedure has been used to account for the observed data into MINNI fields. Specifically, the kriging is applied on the observed data and the external drift is constituted by the MINNI model output. Observations were interpreted as realizations of a Gaussian spatial process *Y(s)* at spatial location *s*, in the domain *S*. *Y(s)* has the following structure:

Y(s)=μ(s)+w(s)+ε(s),

where in the trend component *μ(s)* the MINNI model output is introduced, *w(s)* is a stationary Gaussian random process and *ε(s)* is the error term. Regarding observed data introduced in the dispersion model outputs, we retrieved data from other Italian regional environmental agencies out of BRACE database (national system: http://www.brace.sinanet.apat.it/). Only background stations, whose spatial representativeness is consistent with the MINNI resolution, and only monitoring sites with more than 80% of data have been considered and averaged to obtain annual observed values [11].

PM2.5 and NO_2_ are the most impacting pollutants on the Italian territory, in terms of long-term effect on human health. Also, SO_2_ and CO have historically been of high impact, but their airborne levels have been drastically reduced before year 2000, therefore their impact is very low in the time span analyzed in this study. Black carbon was included in official EU emission inventories in 2017, so no national emission data were available at the time of this study.

Since the health data from the NHIS are aggregated at the municipal level, the exposure assessment must rely on the same spatial scale. Therefore, following this data fusion approach, the Italian exposure assessment was carried out by up-scaling the gridded data at the municipality level [10]. These annual exposure maps were obtained overlying gridded concentration data to municipality boundaries and then using a weighted average, where built-up area percentages were the weights. Built-up area information has been collected from Corine Land Cover data [12] using land use classes belonging to Artificial Surfaces categories. In this way, for both pollutants a mean annual value has been calculated for all Italian municipalities (Fig. 1).

The change of support problem was solved by means of an upscaling technique, since health data are at municipality level, a scale (blocks) larger than the spatial scale of model calculation, which consists of points regularly spaced on a grid.

Since our spatial domain was discretized by means of grid points (or cells), we identified the ni points belonging to the ith municipality employing geographic information system (GIS) software. Therefore, we implemented a weighted block averaging procedure that can be written as

$$C_{i}=\sum_{p=1}^{n_{i}}\gamma_{ip}y_{p}$$

where C_i_ denotes the concentration mean on the ith municipality, y_p_ represents the concentration value of the pth cell and γ_ip_ is the weight for the pth grid point of the ith municipality.

The weight was chosen as

$$\gamma_{ip}=\frac{A_{ip}}{A_{i}}$$

where A_ip_ denotes the municipality built-up area belonging to the pth cell and Ai the whole municipality built-up area. This kind of weight was chosen since built-up areas are associated with anthropic activities and so represent part of the municipality where most people spend their time, including residential and working areas.

**Fig. 3:**
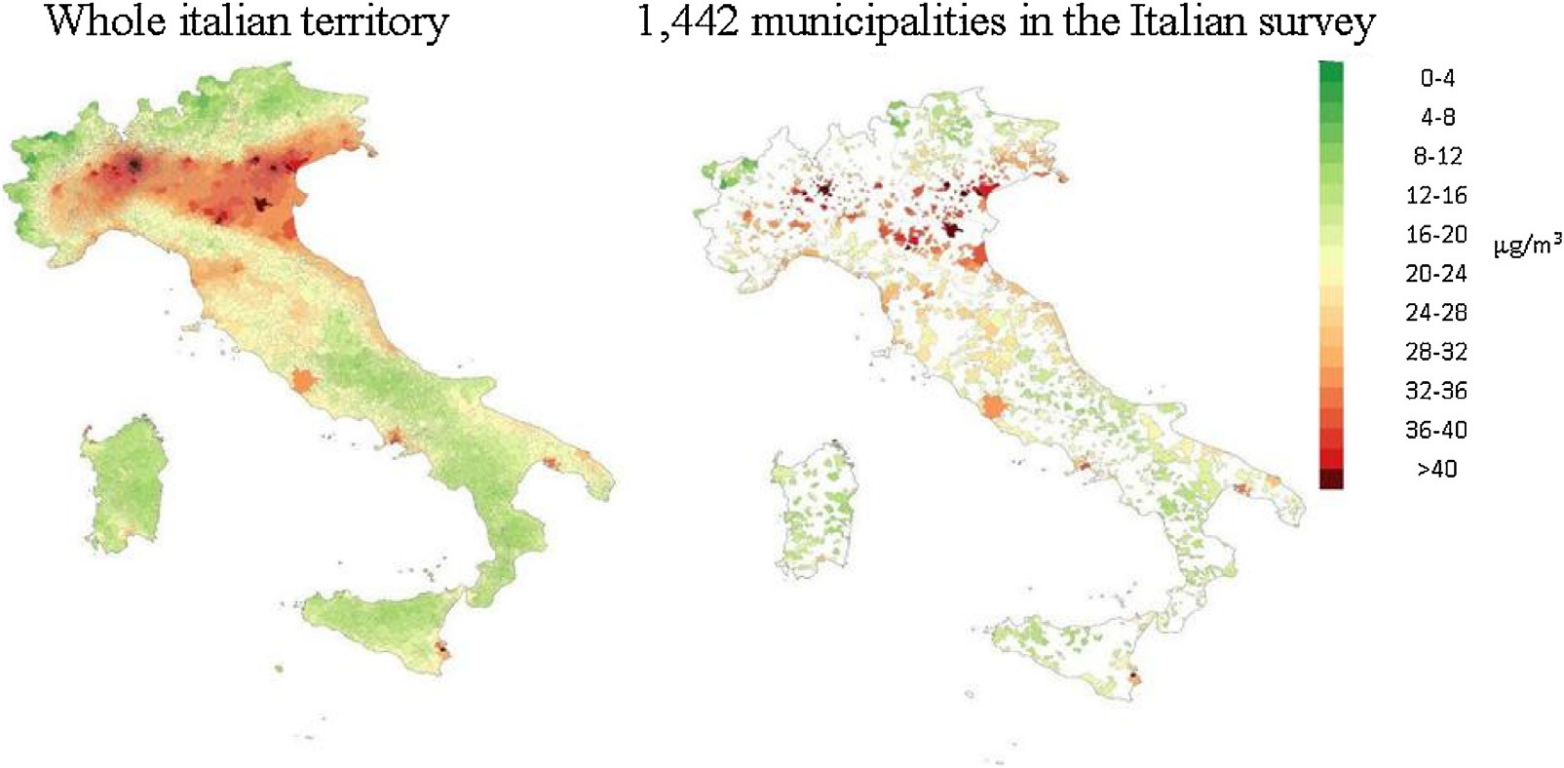
Example of one year exposure assessment – PM2.5 year 2005.

In Fig. 3 there in an example of one year of exposure assessment (2005), considering PM2.5 as pollutant.

### Statistical analyses

For each outcome analysed, the individual effect of long-term exposure to air pollution on mortality and hospitalization is modelled using the Cox proportional hazard model, with air pollution levels and age class as time-varying variables, while adjusting for other variables. Robust variance estimates are produced to take into account the two-stage sampling strategy used by ISTAT (Istituto Nazionale di Statistica) in the survey (sampling municipalities at the first stage and households within each municipality at the second stage, and enrolling all the individuals of the sampled families).

We estimate hazard ratios (HRs) adjusted for gender, educational level (classified according to the international standard classification of education (ISCED) and grouped into the four classes: *up to primary,* corresponding to ISCED 0–1, *lower secondary*, ISCED 2 and 3C, *upper secondary,* ISCED 3A and 3B and *post secondary,* ISCED 4–6), marital status (living with partner/not living with partner), occupational status (employed/not employed), smoking habit (current smoker, former smoker, never smoker), physical activity (4 different levels combining information on type and frequency of activity) and BMI. The individual characteristics are known only at the time of the interview. For each subject we define the residence based on type of municipality according to the population size: *metropolitan areas* (municipalities with more than 250,000 inhabitants), *urban areas* (between 20,000 and 250,000 inhabitants) and *rural areas* (municipalities with less than 20,000 inhabitants).

Results for mortality have been used to test the effectiveness of this methodology, comparing HRs with those obtained in the literature.

The idea of LIFE MED HISS project is to obtain the maximum information from sources already available. Since we had four years of air pollution exposure (from 1999 to 2007), which were subsequent to the enrollment of the cohort, we did not find appropriate to use air pollution as time-dependent variable using only the most recent year of exposure, as done in other studies [14]. Therefore, we divided the follow-up period in 4 risk sets and computed the annual exposure value of each risk set as the mean exposure of all the preceding years for which the data was available, as follows:

• 1999–2002 (exposure in 1999)
• 2003–2004 (mean exposure of 1999 and 2003)
• 2005–2006 (mean exposure of 1999, 2003 and 2005)
• 2007–2008 (mean exposure of 1999, 2003, 2005 and 2007)

Sensitivity analyses have been done using only one year of exposure (2005).

The proportional hazard assumption was tested for all the fixed predictors and stratified Cox models were applied for predictors that did not meet the assumption.

For each outcome, we evaluated potential effect modification by adding into the model the proper interaction term of exposure by modifier. We used the likelihood ratio test to compare the models with and without interaction terms.

Due to the heterogeneous spatial coverage of monitoring stations in Northern and Central Italy (dense spatial coverage) and Southern Italy (sparse spatial coverage), sensitivity analyses have been done also restricted to the first domain.

All statistical analyses were conducted using SAS, version 9.4 (SAS Institute, Cary, NC) and R, version 3.3.3.

## Advantages and disadvantages of the application of the method

Method developed in MED-HISS project aimed at contributing to the updating and development of European Union environmental policy and legislation, in term of adverse health effect of air pollution. The current understanding of the association between long-term exposure to air pollution and adverse health effect is based on cohort studies from USA, Canada, Japan, China. Few studies have been conducted so far in Europe, with restrictions on age of cohort recruited, pollutant studied, and with poor geographical variability. The aim of MED-HISS methodology is to set up a European surveillance system based on retrospective cohorts recruited using National Health Interview Survey (NHIS) data, which are compulsory in European Union, and therefore already available. Cohorts have been followed-up for mortality and hospital admissions. In this study we used the cohort to study long term effects of air pollution. To each subject will be assigned the exposure to air pollution (PM2.5 and NO2), derived from national dispersion models.

The major advantages are:

- it is a low-cost approach if compared with the other studies in this field, which recruited people specifically for the study. To set up a cohort implies the costs to select the sample and fill out questionnaire, which with MED HISS methodology are covered by the institutions which collect data for NHIS
- in this way the cohorts are representative of all populations (urban, rural and metropolitan areas), spread over the whole national territory and with individual chacteristics (e.g. education, working conditions, lifestyle) representative of the whole population
- it can be used for other environmental issues, not necessarily only to study health effect of air pollution
- variables concerning individual characteristics can be easily standardized across countries, as they come from European NHIS.

The disadvantages are:

- it can be restricted due to privacy regulation. For example, in Spain and in Slovenia, it was not possible to apply this method within time table of LIFE MED HISS project. In Spain, the identificative keys of the subjects involved in the NHIS have been destroyed, so that the linkage has not been possible. However, this problem has been brought to the attention of the legislation, succeeded in the linkage with health outcomes with the last Slovenian NHIS (but to study air pollution effect the years of follow-up have not been enough).
- variables concerning individual characteristics are collected only at baseline and cannot be updated (particularly for those who had not experienced the events)

## Additional information

We discourage the application of this methodology to both PM10 and PM2.5. In our experience, due to the attribution of a mean annual value to a whole municipality, the two components of particulate matter are highly correlated. Instead, if it is possible to go in depth within municipality with information of subjects’ address, this issue should not be a problem. Moreover, particularly in years 1999 and 2003, assimilation procedure for PM2.5 has been done deriving the monitoring stations data from PM10.

The sensitivity analysis with only 2005 annual mean as exposure is due to a low spatial coverage of monitoring stations for the year 1999.

Although it has been possible to recruit the cohort and an extension of the follow-up will be available, we had few limitations as well:

• The impossibility to get home address or zip code, which would have been ensured a higher degree of accuracy in exposure assessment
• The impossibility to have information on residential history (we only had the information at baseline)

The number of years of follow-up depends on the topic and not on the methodology. To study long-term effect of air pollution we needed several years from the time of the interview to evaluate the health effect of air pollution on the outcomes under investigation.
